# Single Rapamycin Administration Induces Prolonged Downward Shift in Defended Body Weight in Rats

**DOI:** 10.1371/journal.pone.0093691

**Published:** 2014-05-02

**Authors:** Mark Hebert, Maria Licursi, Brittany Jensen, Ashley Baker, Steve Milway, Charles Malsbury, Virginia L. Grant, Robert Adamec, Michiru Hirasawa, Jacqueline Blundell

**Affiliations:** 1 Department of Psychology, Memorial University of Newfoundland, St. John's, Newfoundland, Canada; 2 Division of Biomedical Sciences, Memorial University of Newfoundland, St. John's, Newfoundland, Canada; CRCHUM-Montreal Diabetes Research Center, Canada

## Abstract

Manipulation of body weight set point may be an effective weight loss and maintenance strategy as the homeostatic mechanism governing energy balance remains intact even in obese conditions and counters the effort to lose weight. However, how the set point is determined is not well understood. We show that a single injection of rapamycin (RAP), an mTOR inhibitor, is sufficient to shift the set point in rats. Intraperitoneal RAP decreased food intake and daily weight gain for several days, but surprisingly, there was also a long-term reduction in body weight which lasted at least 10 weeks without additional RAP injection. These effects were not due to malaise or glucose intolerance. Two RAP administrations with a two-week interval had additive effects on body weight without desensitization and significantly reduced the white adipose tissue weight. When challenged with food deprivation, vehicle and RAP-treated rats responded with rebound hyperphagia, suggesting that RAP was not inhibiting compensatory responses to weight loss. Instead, RAP animals defended a lower body weight achieved after RAP treatment. Decreased food intake and body weight were also seen with intracerebroventricular injection of RAP, indicating that the RAP effect is at least partially mediated by the brain. In summary, we found a novel effect of RAP that maintains lower body weight by shifting the set point long-term. Thus, RAP and related compounds may be unique tools to investigate the mechanisms by which the defended level of body weight is determined; such compounds may also be used to complement weight loss strategy.

## Introduction

The most common weight loss strategy is caloric restriction and exercise, as obesity is typically due to chronic excess in caloric intake over energy expenditure [1]. However, weight loss is strongly countered by physiological compensatory responses that often defeat attempts to stay on a diet regimen and maintain weight loss [2], [3]. It has been proposed that obesity is not a state where energy homeostasis is dysregulated, but where the defended body weight level, or set point, is shifted upwards [4]. This is a major obstacle that needs to be overcome if obesity and overeating are to be contained.

Rapamycin (RAP) is an inhibitor of the mammalian target of rapamycin (mTOR). mTOR is a highly conserved serine/threonine kinase that is inhibited by energy deficiency but activated by energy and nutrient signals to promote cell growth through well described pathways (see [5] for recent review). Inhibition of mTOR by daily RAP administration reduces both food intake and body weight gain in free-feeding animals and provides resistance to diet-induced obesity [6], [7], [8]. In hypothalamic neurons that regulate energy balance and food intake, mTOR has been shown to mediate the anorexic and orexigenic effects of leptin and ghrelin, respectively. These effects can be blocked by direct injections of RAP into these areas [9], [10], [11]. Thus, peripherally administered RAP could exert actions either peripherally or centrally, or both.

In the present study, we examined the effect of a single injection of RAP (peripheral or central) on eating and body weight. Consistent with chronic administration [6], [7], [8], acute RAP produced a dose-dependent reduction in both food intake and body weight gain. Unexpectedly, however, RAP treated animals voluntarily maintained a lower body weight for weeks and months in the absence of additional RAP administration. The persistent lowered body weight by RAP could be explained by a sustained downward shift in body weight set point or a disruption of compensatory mechanisms for regaining body weight. Thus, the goal of the current study was to test the hypothesis that acute RAP causes a downward shift in body weight set point. Overall, our findings suggest a novel role of mTOR in *establishing* a homeostatic set point and that RAP may be a unique tool for probing the determinants of body weight set point.

## Methods

### Animals

Male Sprague Dawley rats were obtained from the Vivarium at Memorial University of Newfoundland at 7 weeks of age. The rats were housed individually in a temperature- and humidity-controlled environment with a 12-h light/12-h dark cycle (lights on at 7:00 am). Rats were given free access to a standard rodent diet (Prolab RMH 3000: PMI Nutrition International LLC, Brentwood, MO, USA) and water, unless otherwise stated. Body weight and food intake were measured every 1–2 days unless indicated otherwise at the same time each day (9:00–11:00 am).

### Ethics Statement

All procedures involving animals adhered to the guidelines of the Canadian Council on Animal Care, and were approved by the Institutional Animal Care Committee of Memorial University.

### Rapamycin injection

For intraperitoneal (i.p.) administration, rats received either vehicle (VEH: 5% ethanol in 5% Tween 80 and 5% PEG 400 in distilled water) or RAP (LC Laboratories, Woburn, MA, USA) in vehicle at 0.1, 1, or 10 mg/kg, similar to [12], [13].

For intracerebroventricular (i.c.v.) injection, rats were initially implanted stereotaxically with guide cannulae aimed at the left lateral ventricle under 4% chloral hydrate (400 mg/kg i.p.). After 16–19 days of recovery, rats received an i.c.v. injection of 1 µL of DMSO as vehicle (VEH-ICV) or 50 µg of RAP in 1 µL DMSO (RAP-ICV), similar to [9]. Upon completion of the experiment, rats were anesthetized with 15% urethane and brains were collected. To verify location of cannula tips, brains were sectioned and stained with cresyl violet and examined microscopically. Tracks formed by the guide cannulae reached the lateral ventricle in all subjects.

### Visceral Fat

Visceral fat was assessed in a sub-set of subjects that received 2 injections a week apart of VEH or RAP (RAP-RAP and VEH-VEH groups). Rats were killed by CO_2_ inhalation approximately 2 weeks following the second injection. Retroperitoneal and epididymal fat pads were dissected and weighed immediately to determine total visceral fat mass.

### Glucose Tolerance Test

Two groups of rats matched by weight were injected i.p. with RAP (10 mg/kg) or VEH. Two weeks later, the rats were fasted overnight for 16 hours. To establish basal values of blood glucose (fasted), a drop of blood was drawn by nicking the tail vein with a razor blade and glucose level in whole blood was measured with Blood Glucose Monitoring System (Free Style Lite, Abbott). Then the rats were injected with glucose solution (in H_2_O, 2 g/kg i.p., Time 0). Blood glucose levels were measured at 15, 30, 60, 90 and 120 minutes post-injection.

### Conditioned Taste Aversion Test

All rats had unrestricted access to rodent chow and restricted access to water (one hour each day, 9:00–10:00 am) for one week (Days 1–7) prior to injection. Body weights were measured approximately 2 hours later each day. On injection day (Day 8), all rats were presented with one bottle containing 0.1% saccharin in water for one hour (between 9:00–10:00 am). Immediately following saccharin consumption, rats received an i.p. injection of RAP (10 mg/kg), VEH, LiCl (as a positive control, dose of 127.17 mg/kg), or saline (vehicle for LiCl). The next day (Day 9), the rats had 1-hour access to water. On Day 10, the rats were given a 1-hour two-bottle preference test during which they had access to a bottle containing 0.1% saccharin solution and another bottle containing water. At 30 minutes into the test, the places of bottles were exchanged to control for side preference effects.

Saccharin preference was calculated as a ratio of the total amount of saccharin consumed during the one hour period to the total amount of fluid (water + saccharin solution) consumed. A a lower saccharin preference measure from controls indicates whether the drug has produced a conditioned aversion to the associated saccharin.

### Yoke Procedure

Rats were divided into three groups having approximately equal baseline food intake (differed by less than 1 gram). Rats in two of the groups were ranked by food intake from highest to lowest. Pairs were formed by taking the two subjects with the highest food intake, the two with the next highest food intake, and so forth. Within each pair one animal was randomly assigned to the RAP and the other to the yoked condition (YOKE). The third group formed the VEH group. Following the five day baseline period, rats were injected i.p. on Day 0 with RAP (10 mg/kg) or vehicle (VEH and YOKE groups). During the yoked period, daily food intake was determined for each rat in the RAP group and expressed as a percentage of its averaged daily food intake during the baseline period. Each day of the 5-day yoked period, yoked rats were given a percentage of their daily baseline food intake amount which corresponded to that of their RAP counterpart for the preceding 24 hour period. Animals were then placed back on ad lib food; daily food intake and body weight were measured for an additional week.

### Food deprivation

Rats were given either a RAP (10 mg/kg) or VEH injection i.p., and then 24 hours or 2 weeks later, food was restricted to 5 grams for 24 hours. All rats were returned to *ad libitum* feeding following deprivation for the remainder of the experiment. Water was available *ad libitum* at all times.

### Data analysis

All body weights were expressed as a percentage of injection day (Day 0) body weight in order to adjust for individual differences in absolute body weight. Food efficiency (FE) was calculated by dividing body weight gain by food intake (FI) (both in grams) for each 24 hour period. There were no pre-treatment differences among the groups in weight gain, FI or FE in any of the experiments. One-way ANOVAs with Tukey's post hoc tests were used to test for differences among three or more groups, whereas unpaired t-test was used to compare two groups, as appropriate. Two-way Mixed ANOVA was used to compare two or more groups that were repeatedly measured to follow the time course. Paired t-test was used for within-group comparisons. Data are expressed as mean ± S.E.M. *p*<0.05 was considered significant.

## Results

### Single injection of rapamycin inhibits food intake and body weight gain

**Figure 1:**
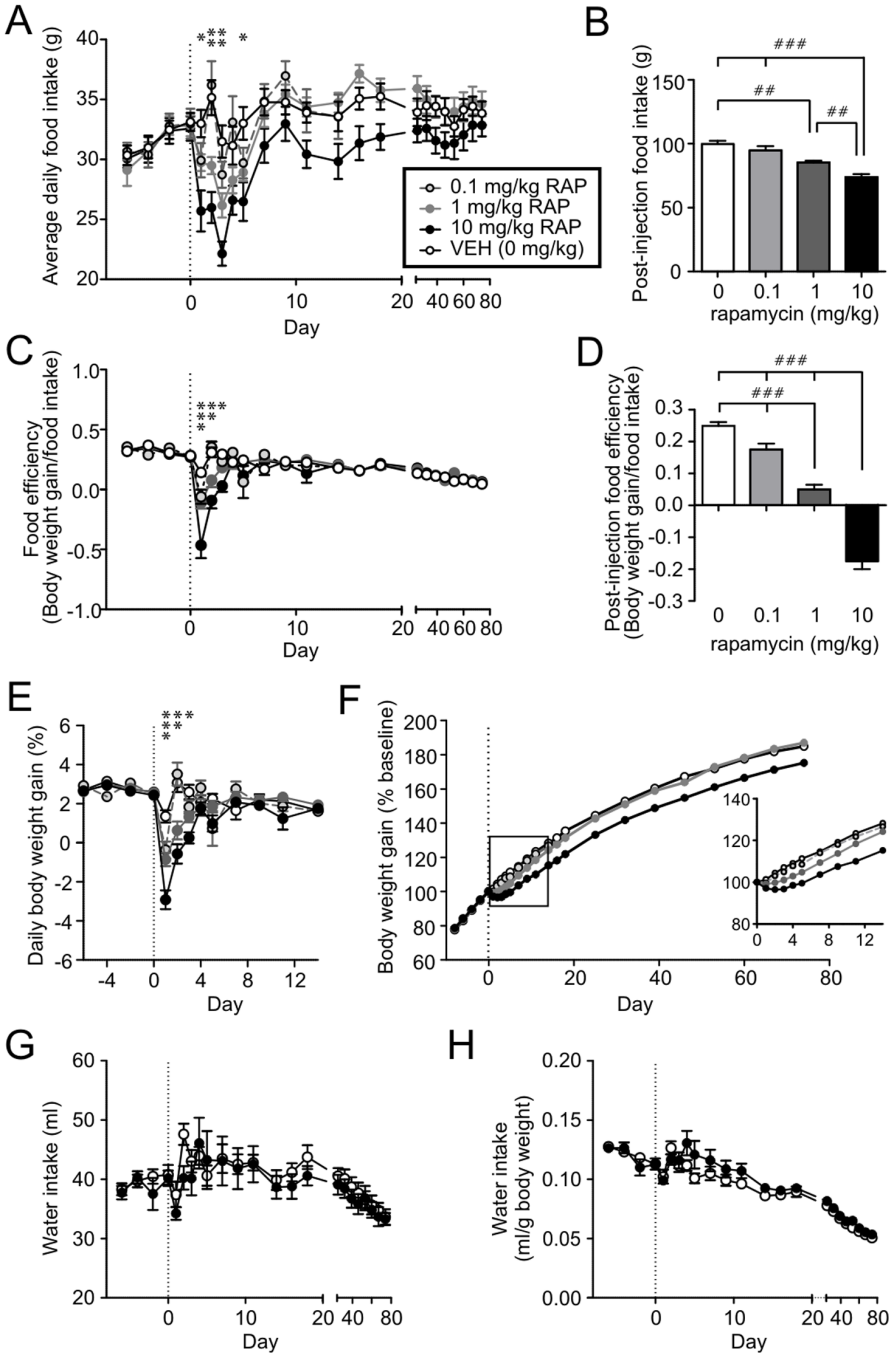
Single systemic injection of rapamycin induces prolonged decrease in body weight gain. A: Rapamycin (RAP) i.p. injection on Day 0 (vertical broken line) induces a transient decrease in daily food intake. B: Three-day cumulative food intake (Day 1–3 post-injection) shows a dose-dependent inhibition. C: RAP induces a transient decrease in food efficiency. D: Three-day cumulative food efficiency (Day 1–3) shows a dose-dependent suppression. E: RAP induces a transient decrease in daily weight gain. For panel A, C and E, *p<0.001 for 10 mg/kg vs.VEH; **p<0.05 for 1 and 10 mg/kg vs. VEH; ***p<0.05 for all RAP doses vs.VEH (two-way Mixed-ANOVA). For panels B and D, ##p<0.01, ###p<0.001 (one-way ANOVA with Tukey's test). F: Cumulative body weight gain curve depicting that RAP injection results in a downward shift in body weight. The first 2 weeks (box) is expanded and shown in the inset. The effect is dose-dependent. VEH vs.10 mg/kg, p<0.01 on Day 3–74; VEH vs. 1 mg/kg, p<0.01 on Day 2–11, p<0.05 on Day 14 and 18; VEH vs. 0.1 mg/kg, not significant (two-way Mixed-ANOVA). G, H: Averaged daily water intake (H) and water intake normalized to/body weight (G) shows no difference between RAP (10 mg/kg)-treated animals compared to VEH (two-way Mixed-ANOVA).

To examine the effect of acute RAP treatment on energy balance, 40 male Sprague Dawley rats were given a single i.p. injection of either 0 (vehicle, VEH), 0.1, 1.0 or 10 mg/kg of RAP (n = 10 for each group). We found that food intake (FI) and food efficiency (FE) were significantly reduced during the first 3–5 days post-injection in a dose-dependent manner (Fig. 1A–D). The effect of 10 mg/kg RAP on FI was observed as early as Day 1 post-injection, while the response was delayed to the second day at 1 mg/kg. This was accompanied by a transient (2–3 days) decrease in daily body weight gain, which subsequently returned to baseline levels in all groups (Fig.1E). As a result, the difference in cumulative body weight gain persisted for up to 14 and 74 days for 1 mg/kg and 10 mg/kg, respectively (Fig.1F). Despite the persistent decrease in body weight, there was no difference in fluid intake across days (Fig. 1G, H) between VEH and RAP-treated animals. These results indicate that single RAP injection dose dependently induces sustained reduction in body weight.

### Double rapamycin injections has additive effects on energy balance and reduces fat mass

**Figure 2:**
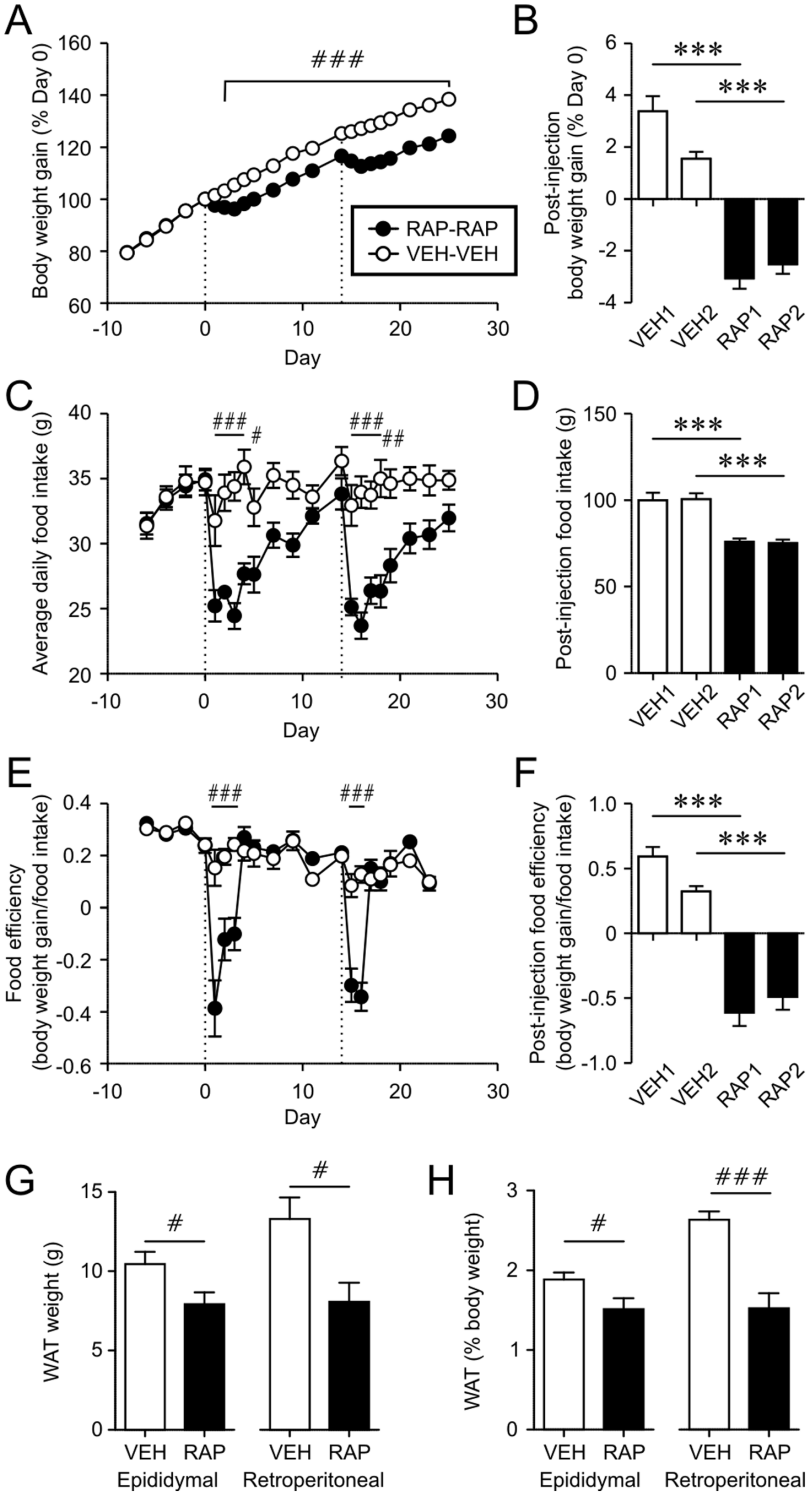
Spaced injections of rapamycin have additive effect on body weight gain. A–C: A, C and E: Body weight gain (A), daily food intake (C) and food efficiency (E) of rats given two i.p. injections (broken lines, Day 0 and 14) of RAP (10 mg/kg each) or VEH with a 2-week interval. # p<0.05, ## p<0.01, ### p<0.001, VEH vs. RAP (two-way Mixed-ANOVA). Horizontal bars indicate the days when significance was seen. B, D and F: Cumulative body weight gain, food intake and food efficiency during the first three days post-injection. ***p<0.001, VEH vs. RAP. There was no statistical difference between two injections within the group (VEH1 vs. VEH2, RAP1 vs. RAP2) (two-way Mixed-ANOVA). G: The weight of white adipose tissues (WAT), epididymal and retroperitoneal pads, in rats treated twice with VEH or RAP. H: WAT weight normalized to the body weight of individual rat. # p<0.05, ### p<0.001 (unpaired t-test).

There are reports of resistance to RAP in other experimental contexts [14]. Thus, we examined whether a RAP administration (10 mg/kg, i.p.) would affect responses to a subsequent RAP treatment. Rats received a pair of RAP or VEH i.p. injections (n = 10 each) with a 2-week interval between injections. We found that the two injections of RAP were equally effective in reducing FI, FE and weight gain (Fig.2A–F). The VEH group showed a tendency of lower weight gain and FE after the second injection compared to the first, which may be due to age-dependent slowing of the rate of weight gain (Fig.2B). In RAP treated animals (n = 6), the white adipose tissues were significantly smaller than those of VEH controls (n = 5) (Fig.2G, H), consistent with previous reports showing decreased adiposity following chronic RAP administration [6], [7]. These data suggest that the RAP effect does not desensitize with intermittent injections, at least when injections are separated by 2 weeks, and it effectively reduces adiposity.

### Possible side effects of single RAP injection

**Figure 3:**
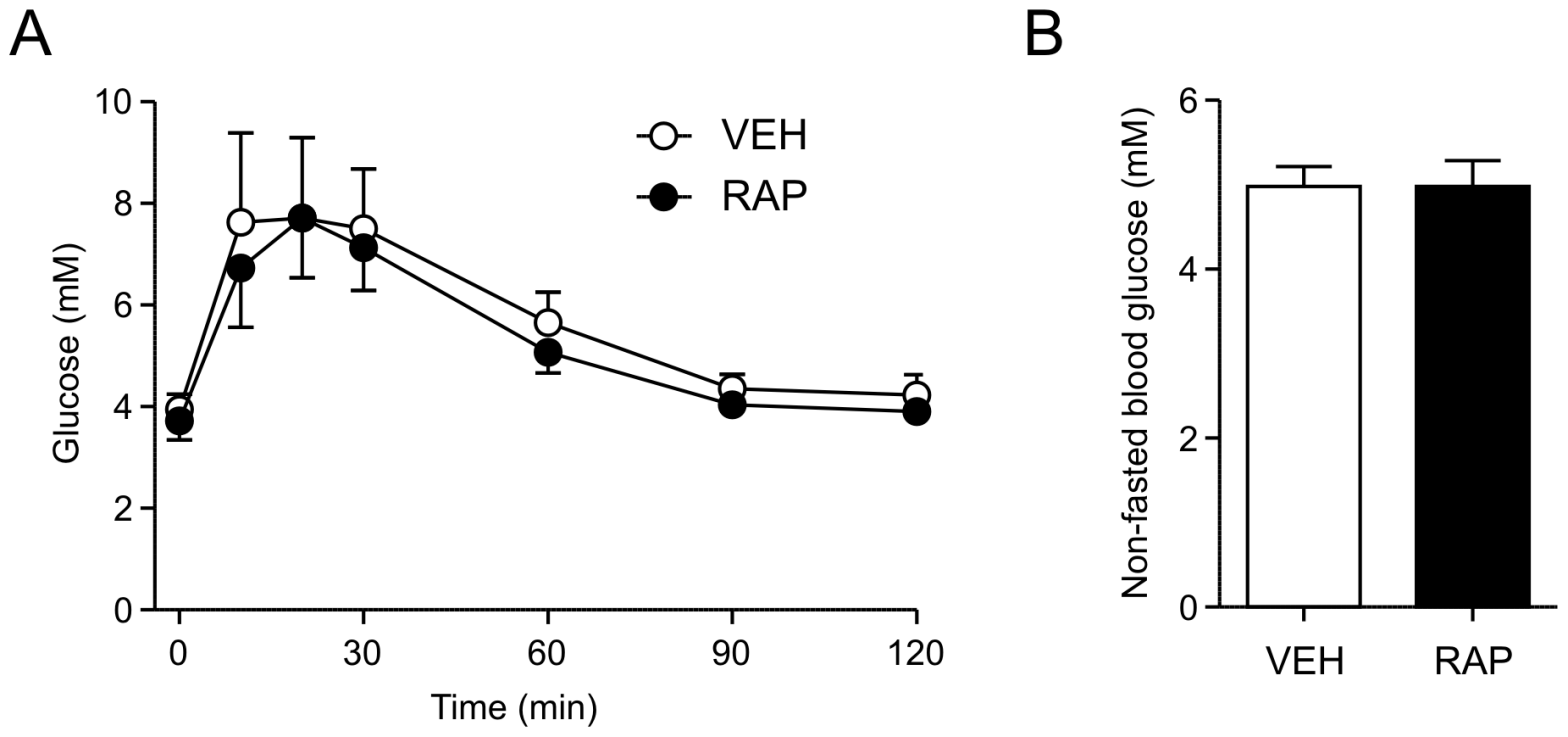
Rapamycin does not affect glucose tolerance. A: There were no differences in blood glucose levels measured at 15, 30, 60, 90 and 120 minutes post-glucose injection in RAP and VEH animals (two-way Mixed-ANOVA). B: There is no difference in non-fasted blood glucose in rats administered with VEH or RAP (10 mg/kg i.p.) at 2 weeks post-injection (unpaired t-test).

Since chronic RAP administration is known to induce glucose intolerance [6], [15], [16], [17], we conducted a glucose tolerance test two weeks after single injection of RAP (10 mg/kg i.p., n = 8) or VEH (n = 7). There was no difference in the fasting blood glucose or response to glucose challenge between the two groups (Fig.3A). Furthermore, in a separate cohort of animals (n = 5 each), we found no difference in non-fasting blood glucose levels at 2-week post-injection (Fig.3B). Therefore, a single RAP injection does not appear to influence glucose homeostasis long-term, unlike the glucose tolerance that develops with daily administrations of RAP over a 2-week period, as shown previously [6], [15], [16], [17].

**Figure 4:**
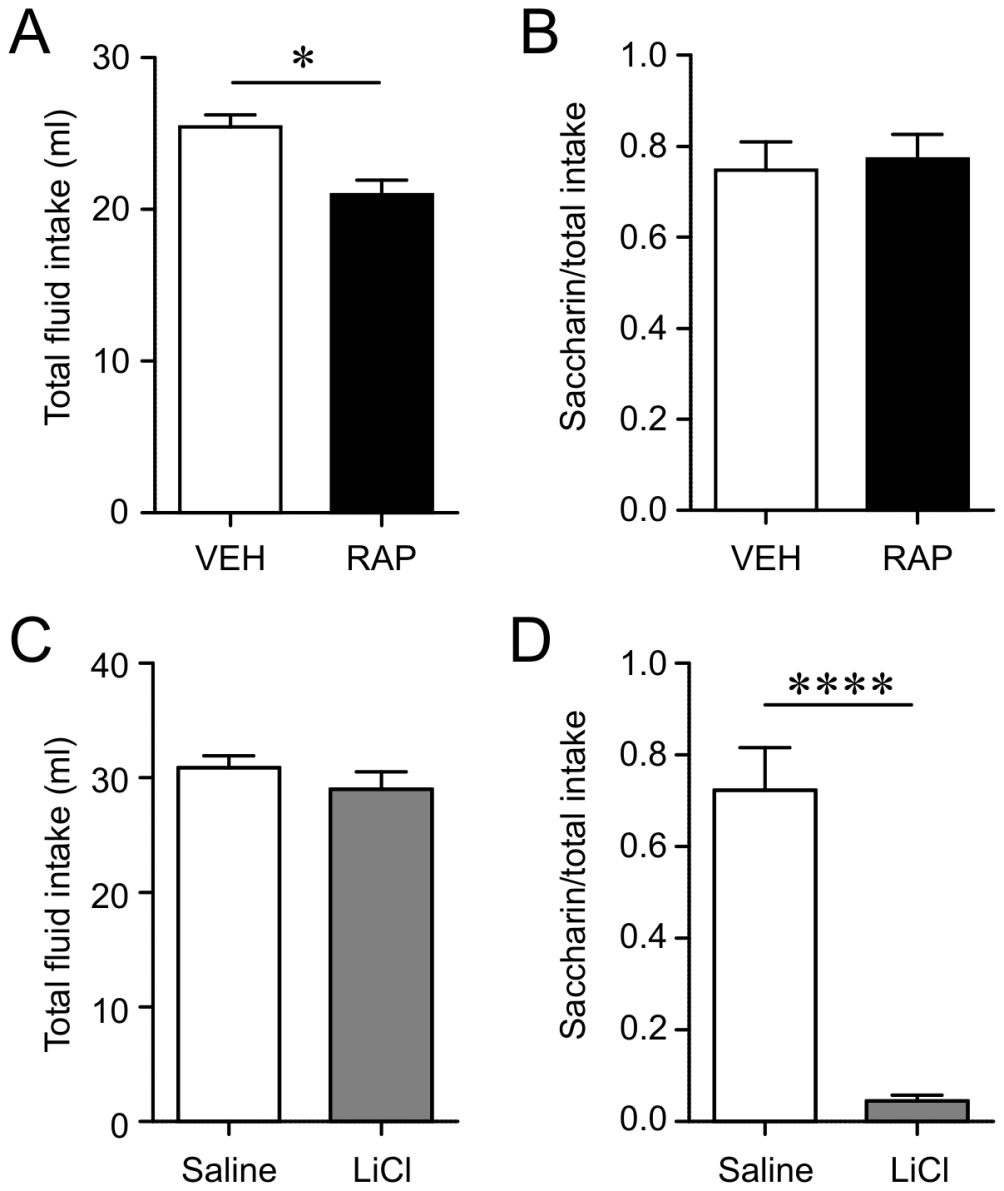
Rapamycin does not induce malaise or illness. A: During the two-bottle test, RAP group ingested significantly less fluid (sum of water and 0.1% Saccharin solution). *p<0.05 (unpaired t-test). B: There was no difference in saccharin preference (unpaired t-test). C: During the two-bottle test, there was no difference in total fluid intake in LiCl- and VEH-treated rats (sum of water and 0.1% Saccharin solution). D: LiCl-treated rats showed a significantly lower saccharin preference compared to VEH-treated rats. ****p<0.0001 (unpaired t-test)

There is a possibility that the reduced eating from RAP could be due, at least in part, to sickness induced by the drug. The typical conditioned taste aversion (CTA) procedure provides a robust and sensitive test of drug-induced sickness, where animals are allowed to drink a novel-flavored solution following which they are injected with a drug. If the drug induced sickness, the animals would show a CTA later for that flavored solution. Rats were given 1 hour access to a novel saccharin solution (0.1% in water) followed immediately by either RAP (10 mg/kg i.p., n = 9) or VEH (n = 9). A two-bottle choice test was administered 2 days later when the rats were given simultaneous access to water and 0.1% saccharin solution. The total fluid intake from saccharin solution and water was greater in VEH than RAP rats (Fig. 4A). This is in contrast to the lack of effect of RAP on water intake (Fig. 1G, H), which may be due to the experimental condition of the CTA test, involving restricted fluid access and a choice of water and saccharin solution. Saccharin preference was calculated as the proportion of saccharin solution intake over total fluid intake. This test indicated no differences in saccharin preference between RAP and VEH treated rats (Fig.4B). In contrast, LiCl (i.p., n = 5) induced a robust decrease in saccharin preference compared to saline injection (n = 5; Fig. 4D), as expected [9]. Thus there was no evidence that RAP induced CTA, which suggests that RAP-induced anorexia is not due to illness.

### Rapamycin lowers the defended level of body weight

**Figure 5:**
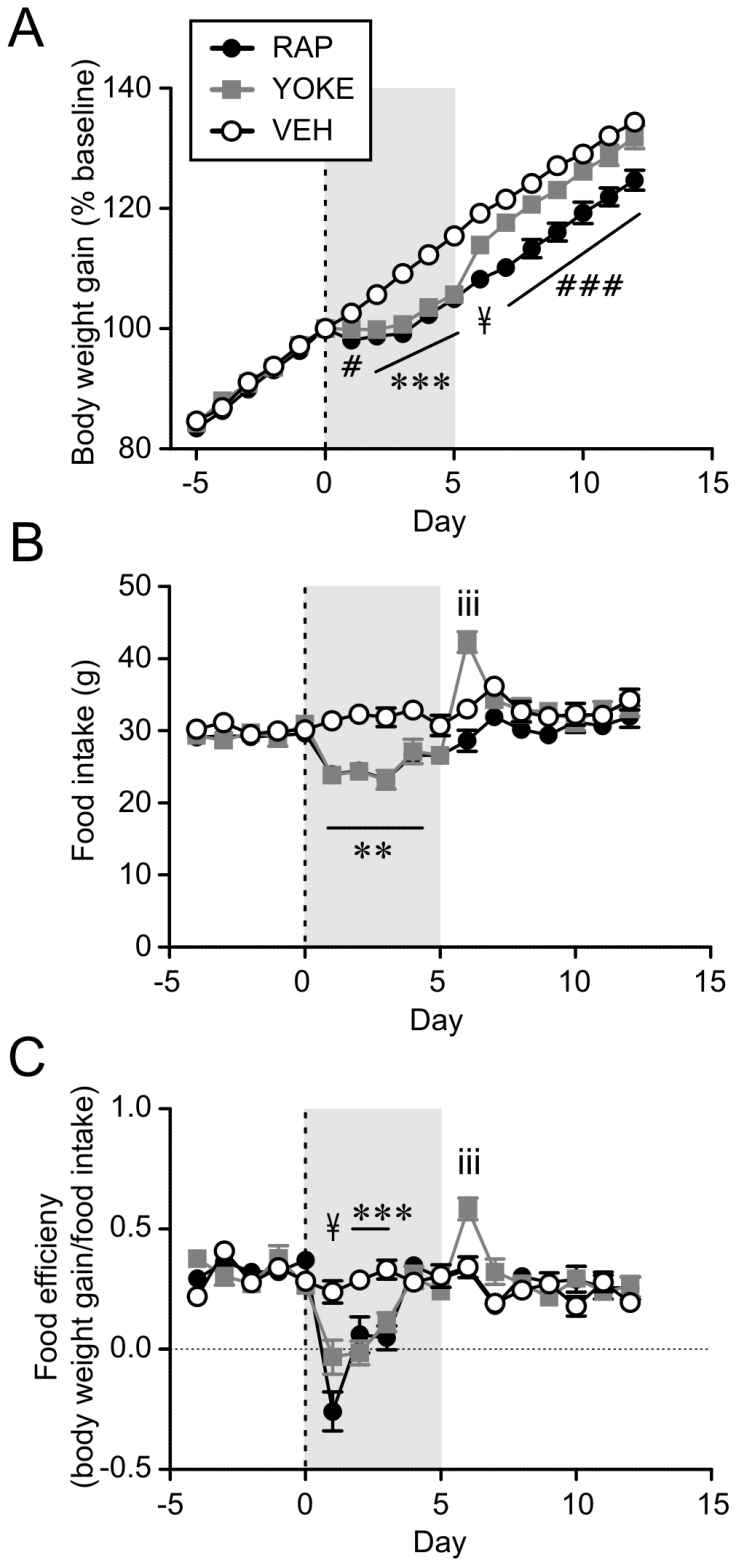
Rats pair-fed with RAP-treated animals show compensatory overfeeding and weight rebound. A: During the pair-feeding (shaded area), YOKE and RAP groups had lower weight gain compared to VEH group. YOKE group regained weight upon returning to *ad libitum* feeding. B and C: YOKE group show a transient increase in food intake (B) and efficiency (C) following pair-fed period. *p<0.05, ***p<0.001 VEH vs. YOKE and RAP; iii p<0.001 YOKE vs. VEH and RAP; ###p<0.001 RAP vs. VEH and YOKE; #p<0.05 VEH vs.RAP; ¥ p<0.05 all three groups are different from each other (two-way Mixed-ANOVA).

Normally, caloric restriction and/or weight loss are followed by rebound hyperphagia and increased efficiency in food storage. However, RAP-treated animals did not display hyperphagia and body weight remained lower than controls following the acute anorexic phase. This may be explained by the RAP-induced reduction in FI and body weight not being sufficient to engage counter-regulatory responses. To test this idea, a group of rats (YOKE) were pair-fed to match the daily FI of RAP-treated rats for the 5-day period following injection, but otherwise fed *ad libitum*. As expected, the RAP and YOKE groups had lower body weight gain and FI during the pair-feeding period compared to free-feeding VEH treated rats (n = 8 each, Fig.5A, B). RAP and YOKE groups did not differ in body weight during this period (p>0.05). However, the YOKE group displayed an immediate rebound in FI and FE during the first day upon returning to free-feeding following the yoked period (Fig.5B, C). This suggests that the degree of anorexia and weight loss induced by RAP is sufficient to activate a counter-regulatory response in non-RAP treated animals.

**Figure 6:**
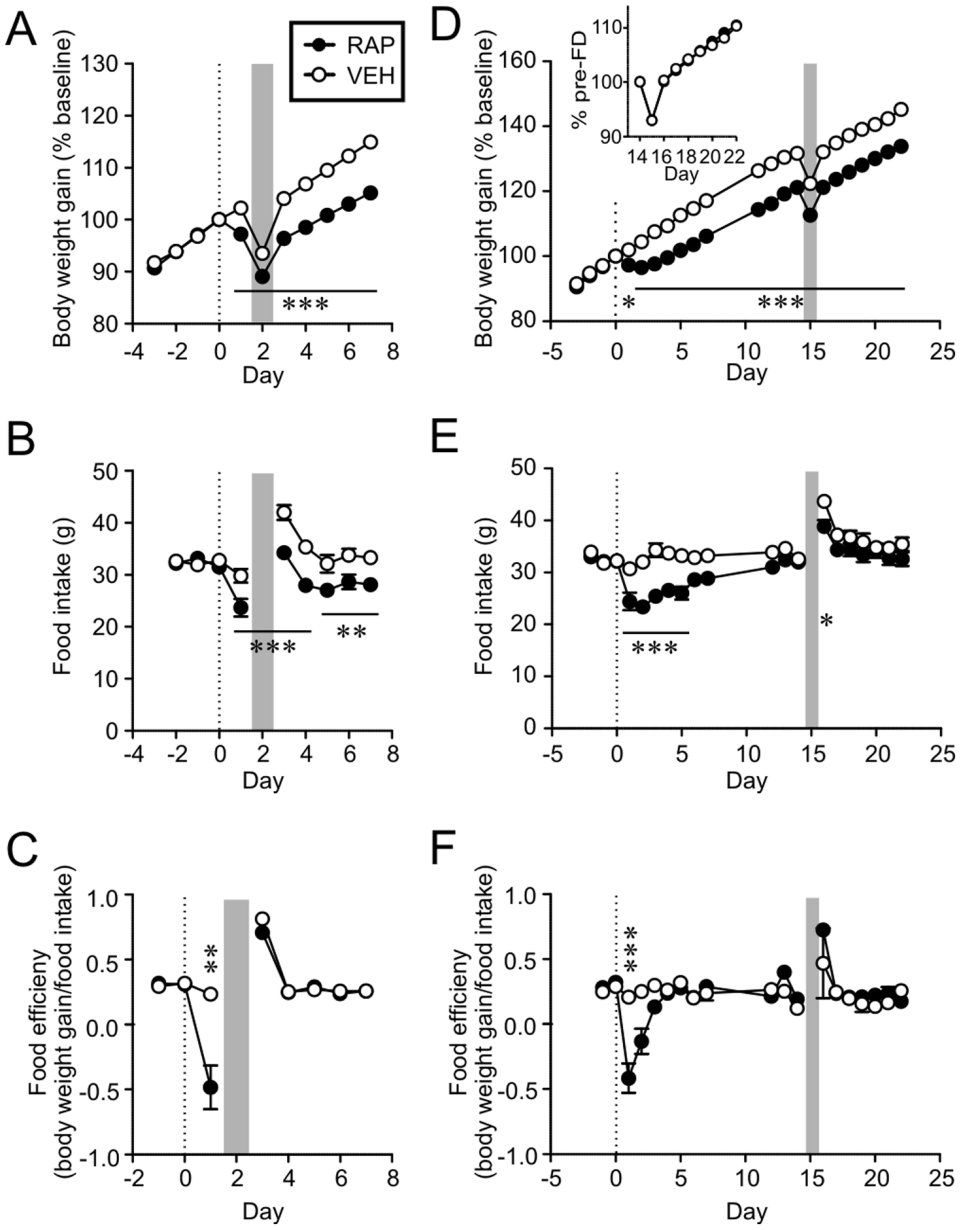
Rapamycin-treated animals defend lower body weight in response to acute perturbation in energy balance. A, B and C: Rats were injected i.p. with RAP or VEH on Day 0 (broken line), and then 24 h later food deprived (FD) as indicated by shaded area. In both groups, FD resulted in an immediate decline in body weight (A), followed by a transient increase in food intake (B) and food efficiency (C) upon refeeding. D, E and F: Rats were injected i.p. with RAP or VEH, then 2 weeks later challenged with FD (shaded area). D inset: Recovery rate of body weight following FD is identical. *p<0.05, **p<0.01, ***p<0.001 (two-way Mixed-ANOVA). Horizontal bars in panel A, B, D and E indicate statistical significance at all time points labeled.

Next, we sought to determine whether RAP prevented the development of compensatory responses to transient reduction in FI and weight. To do this, rats were treated i.p. with either RAP (10 mg/kg) or VEH, and then challenged with 24 h-food deprivation (FD), beginning at either 24 h or 2 weeks post-injection (immediate or late FD, respectively). At 24 h, acute effects of RAP are present, whereas at 2 weeks the acute effects would have subsided and only long-term effect on body weight remains. Immediate FD induced a transient drop in body weight in both RAP and VEH groups (n = 10 each, Fig.6A). Upon re-feeding, rats recovered their body weight. However, RAP rats settled to a lower weight level than the VEH controls. Both groups showed a significant increase in FI post-FD compared to their respective pre-FD levels (p<0.001, paired t-test, Fig.6B). The magnitude of the increase was similar between groups (VEH 12.2±1.1 g, RAP 10.6±1.7 g; p>0.05, unpaired t-test). FE was also increased, although the RAP group showed a greater change in FE compared to the VEH group due to reduced FE on Day 1 post-injection (i.e. the day before FD; Fig.6C).

When FD was imposed 2 weeks after injection (late FD), both RAP and VEH groups showed a transient decrease in body weight (n = 10 each, Fig.6D), which recovered at an identical rate (Fig.6D inset). There was a transient increase in FI and FE during re-feeding in both groups (Fig.6E, F). The increase in FI (pre- vs. post-FD) was less pronounced in the RAP group (VEH 11.1±1.0 g, RAP 6.8±0.7 g, p<0.005, unpaired t-test), which is likely due to the difference in absolute body weight, as there was no difference in FI normalized to body weight (VEH 0.100±0.002, RAP 0.098±0.003, p>0.05, unpaired t-test). These results strongly suggest that RAP-treated animals are capable of activating compensatory mechanisms to defend their body weight in response to acute perturbations, even during the early phase post-injection when rats do not attempt to recover the weight loss by RAP. Therefore, it appears that RAP does not simply inhibit FI and FE, but rather lowers the defended level of body weight.

### Intracerebroventricular rapamycin produces prolonged weight reduction

**Figure 7:**
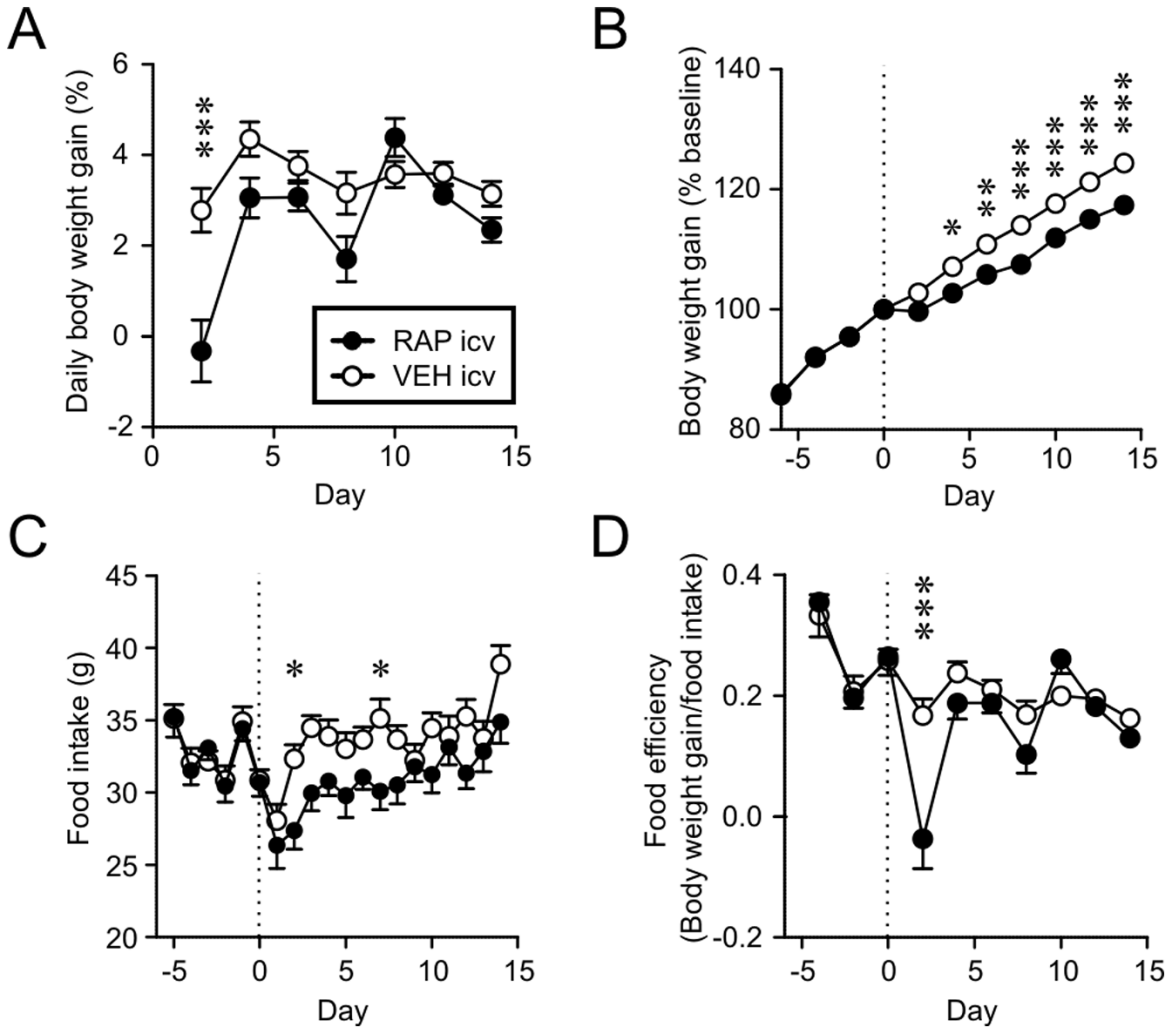
Rapamycin acts in the brain to decrease body weight gain. A and B: RAP i.c.v. injection (Day 0, broken line) induces a transient decrease in body weight gain (A), which results in prolonged shift in body weight (B). C: Food intake is inhibited by RAP i.c.v. D: Food efficiency is inhibited by RAP i.c.v. *p<0.05, **p<0.01, ***p<0.001 (two-way Mixed-ANOVA).

To determine whether the effect of systemic RAP that we observed was due to a central action, we conducted a central injection study. RAP i.c.v. (n = 9) reduced the daily weight gain, FI and FE transiently (Fig.7A,C,D) and cumulative weight gain for up to 15 days compared to those that received equal volume (1 µL) of VEH i.c.v. (n = 11) (Fig.7B). There was some delay in the effect; suppression of FI and cumulative weight gain became significant on Day 2 and Day 4 post-injection, respectively. This is similar to the delayed response seen after 1 mg/kg i.p. injection. These results suggest that the effect of RAP is at least partially mediated by the brain.

## Discussion

The present study shows that single injection of RAP induces a transient decrease in FI, FE and daily weight gain lasting for several days. Surprisingly, the lowered body weight persists for at least 74 days. These effects are not likely due to malaise or illness, as there was no evidence for conditioned taste aversion to RAP. Once the transient effects subside and lower body weight is attained, RAP treated animals do not compensate for the lost weight, unlike pair-fed controls who overeat upon resuming *ad-libitum* feeding and regain weight. Instead, the rate of weight gain, FI and FE of RAP rats is restored after 3–5 days to the levels of vehicle controls, suggesting that energy homeostasis is re-established. Subsequently, the RAP-treated animals defend the newly established lower body weight upon acute perturbations in energy balance by adjusting FI and FE. Specifically, acute food deprivation induces a similar rebound hyperphagia upon re-feeding and an identical rate of body weight recovery in RAP and vehicle-treated groups. When food deprivation is applied within the first few days following RAP injection during the transient suppression of FI, rats are still able to respond with rebound hyperphagia. This indicates that the lack of hyperphagia following RAP is not due to a RAP-induced failure to activate counter-regulatory responses. Also this supports our contention that anorexia did not result from illness, as these animals are capable of eating as much as pre-injection levels. Taken together, our data indicate that RAP induces a downward shift in body weight set point, and that the initial transient decrease in FI and FE following injection is a compensatory response to the disparity between the actual body weight and lowered set point. Therefore, our study shows a novel effect of RAP on body weight regulation.

While peripheral action cannot be ruled out, our findings strongly suggest that RAP acts centrally to exert its effect on energy homeostasis. It is known that peripherally administered RAP readily enters the brain [18] and inhibits p70S6K phosphorylation, a downstream substrate of mTOR signaling [19]. While the highest dose tested in our study (10 mg/kg) is higher than those of previous food and body weight studies (0.2–5 mg/kg/day) [6], [7], [8], [10], [20], 1 mg/kg in our study also produced a significant effect on long-term weight gain. Also, the highest dose used here is well below the RAP doses (e.g. 40 mg/kg) that are effective in other experimental contexts such as fear conditioning [12]. The elimination half-life of RAP is relatively long (approximately 30 hours in rats [21]), however, this would not appear to be of sufficient duration to suppress its target molecule for the entire 10-week observation period in the present study. Therefore, it is likely that a transient action of RAP is enough to induce a long-lasting change in the neural circuitry for body weight regulation. This may involve cap-dependent translation regulated by mTOR, which has been implicated in synaptic plasticity [22].

It is well established that mTOR plays an important role in the control of FI. Metabolic signals such as leptin and branched-chain amino acids (e.g., leucine) activate mTOR1 to inhibit FI [9]. Accordingly, a single central injection of RAP has been shown to increase FI transiently in sated rats by acting in the arcuate nucleus and nucleus of the solitary tract in the hypothalamus and brainstem, respectively [9], [23]. This hyperphagic effect is short-lived; it gradually diminishes with time within the first day post-injection. We did not observe any increase in FI following systemic or central injection of RAP, which may be because our earliest time point was 24 h post-injection. The response we observed was delayed by one day in rats treated with 1 mg/kg i.p. or 50 µg i.c.v., which may be explained by hyperphagic and hypophagic responses balancing out during the first 24 h. Alternatively, the discrepancy may arise from the differences in feeding protocol. Previous studies induced satiation prior to RAP injection by overnight fast followed by re-feeding [23] or exposure to palatable food [9]. Under such conditions, neurons that mediate satiety may be fully activated and cannot be stimulated further. Another possibility is an involvement of a mechanism recruited by orexigenic factors such as ghrelin and thyroid hormone, which activates mTOR pathway and agouti related protein/neuropeptide Y neurons in the hypothalamus to induce FI [11], [24]. Single central injection of RAP is sufficient to block the orexigenic effect of ghrelin [11]. Overall, these contrasting effects of mTOR and RAP on FI may involve different brain regions and/or neuronal populations [11], [25].

Chronic RAP administration (daily injections) also has mixed effects on FI, which seems to depend on animal species, age, diet and duration of RAP treatment, although weight gain is consistently inhibited [6], [7], [8], [10], [20]. In aged mice, mTOR promotes positive energy balance by negatively regulating the activity of POMC neurons in the arcuate nucleus, which is reversed by chronic RAP, suggesting an important role of POMC neurons in the central RAP effect, at least with chronic administration [10]. It is likely that at least some of the known effects of chronic RAP treatment overlap with those seen in our acute injection study, and reduced body weight may not be readily reversible even when the RAP treatment is terminated.

Manipulation of set point would be an effective weight loss and maintenance strategy, as deviating from the set point normally results in strong activation of physiological compensatory mechanisms. We showed that acute injection of RAP, which presumably induces a transient suppression of mTOR, can have a long-lasting effect on the set point for body weight, suggesting a novel role of mTOR in body weight regulation. Moreover, a single injection has distinct advantage as it can avoid side effects of chronic RAP administration such as glucose intolerance. We propose that RAP and related compounds could be used as tools to investigate how the defended level (apparent set point) of body weight is determined and to complement other weight loss strategies.
